# Perceived Availability of Culturally and Demographically Diverse Photographs for Health Education Materials, Colorado, 2010

**DOI:** 10.5888/pcd12.140450

**Published:** 2015-02-26

**Authors:** Mary K. Buller, Erwin Bettinghaus, David B. Buller, Xia Liu, Lyndsay Fluharty

**Affiliations:** Author Affiliations: Erwin Bettinghaus, David B. Buller, Xia Liu, Lyndsay Fluharty, Klein Buendel, Inc, Golden, Colorado.

## Abstract

An online survey was conducted with health educators in Colorado to ascertain their needs and ability to access relevant stock art photographs for their print and electronic educational media. Health educators were dissatisfied with the cultural and demographic diversity of photographs available from their own sources or from commercial stock art websites. There was a perceived need for more photographs that would better represent their target populations. The health educators believed, furthermore, that representative visual images can help improve their message effectiveness.

## Objective

The purpose of this survey was to determine whether health educators have difficulty or ease in finding culturally and demographically diverse photographs for their health education materials. For example, could they find photographs of their unique target populations on existing commercial stock art websites? Could they find photographs of their target populations engaging in the healthy or unhealthy behaviors they wish to represent in their materials? Did they believe matching photographs to the target population can improve the effectiveness of the health message? The survey provided guidance for the development of a larger study to test visual images and message effectiveness.

## Methods

During 6 months in 2010, an invitation to participate in an online survey was emailed to 489 public health education organizations. The sample was drawn from public health membership organizations in Colorado such as nonprofit health organizations, public health foundations, rural health centers, and educational health sciences programs. The organizations were asked to forward the survey to the person in their organization responsible for creating health education media. Three weeks after the initial email invitation, a reminder email was sent to all organizations. Exclusions to the survey included being younger than 21 years and not able to read or speak English. No respondents identified as being ineligible, and therefore no surveys were removed from the final analysis. The Inquisite (Millisecond Software, Version 9.6) online survey consisted of 26 questions asking about primary target populations reached, types of health education messages and materials created, and availability of stock photographs needed. Questions were categorical Likert-type scales or open-ended. For example: “On a scale of 1 to 5, how well do the photographs that are available to you match the demographics and physical characteristics of the target populations you are trying to reach with your materials?” Survey material and protocols were approved by the Western Institutional Review Board.

## Results

A total of 151 people completed the online survey (31% response rate). Respondents were 88% female, 5% Hispanic, and aged 24 to 68 years (mean age, 44 y) (Table). Eighty percent of those surveyed indicated that their target populations were low income. Respondents also reported targeting people of a wide range of ages (0–79 y) and racial groups; 89% reported targeting whites; 56%, African Americans; 27%, American Indians/Alaskan Natives; 33%, Asians; and 23%, Pacific Islanders. More than two-thirds (68%) of respondents reported that they developed their own print and electronic material, and nearly all used photographs of people in their print (92%) and electronic (82%) materials. Respondents agreed that matching photographs in educational materials to the target populations improves message attractiveness (62%), grabs attention (68%), makes messages more understandable (50%), and increases relevance (61%) and persuasiveness (68%). Respondents reported that they obtained photographs through external graphic designers (82%), by taking their own photographs (64%), or by purchasing photographs (44%). However, many respondents reported that photographs from commercial stock photography websites were not readily available to match their target populations’ physical characteristics (46%), health conditions and disabilities (57%), common activities (55%), or work/life settings (60%). When asked to rate how well photographs readily available to them (even on a limited basis) matched their primary target population, they reported that photographs they had access to did not adequately match their primary target population on these same features (physical characteristics, 47%; health conditions and disabilities, 55%; common activities, 53%; and settings, 56%). Respondents expressed dissatisfaction with available photographs’ portrayal of demographic diversity (53%), health conditions and disabilities (59%), activities (53%), and settings (56%).

**Table:** Characteristics of Study Population (n = 151), Survey on Cultural and Demographic Diversity in Visual Images, Colorado, 2010

Characteristic	Value^a^
**Sex**	
Male	17 (11)
Female	132 (88)
Unknown	2 (1)
**Hispanic**	
Yes	8 (5)
No	138 (92)
Refused/unknown	5 (3)
**Race**	
American Indian	2 (1)
Asian	5 (3)
African American	7 (5)
White	124 (82)
Other	12 (8)
Unknown	1 (<1)
**Age**	
No. of respondents	149
Mean age, y	44

a Values are no. (%) unless otherwise indicated.

## Discussion

This formative research suggests a need for more diverse images for health education materials. This is consistent with King (1), who recently reported finding few photographs showing subpopulations in a cancer context. Nearly 40% of the US population does not identify as “white alone” (2), more than two-thirds of adults are overweight or obese (3), 15% of the population lives in poverty (2), and the number of adults over 65 is increasing rapidly with the aging of the baby boom generation (4). The rise in the use of the Internet as a health communication vehicle calls for increased use of visual images to attract and hold the attention of all types of people, not just those typically represented on existing photography websites. Results of this study suggest that commercial stock art websites should not be relied on alone to meet the needs of health educators and their diverse target populations. Results also suggest that health educators can take an active role in ensuring the diversity and representativeness of photographs by either taking their own photographs more mindfully or advocating strongly for graphic designers they work with to choose photographs that are more characteristic of the target population. It may take some determination to obtain ideal photographs, but the increase in message impact would be worth the effort. The visual image libraries maintained by the federal government (eg, Centers for Disease Control and Prevention, National Cancer Institute) may be a place to start. This study was limited to public health educators in Colorado, which limits its generalizability, and the percentage of people responding to the questionnaire was smaller than desired. The respondents, however, did represent the people responsible for creating health education materials in their organizations. The results suggest that increased visual representation of the diversity that is the United States will be well received by the health education community.
